# Uncoupling Oncogene-Induced Senescence (OIS) and DNA Damage Response (DDR) triggered by DNA hyper-replication: lessons from primary mouse embryo astrocytes (MEA)

**DOI:** 10.1038/s41598-017-13408-x

**Published:** 2017-10-11

**Authors:** Marcos Seoane, José A. Costoya, Víctor M. Arce

**Affiliations:** Molecular Oncology Laboratory MOL. Departamento de Fisioloxia, Facultade de Medicina and Centro de Investigación en Medicina Molecular e Enfermidades Crónicas (CiMUS). Instituto de Investigación Sanitaria de Santiago de Compostela (IDIS). Universidade de Santiago de Compostela, Santiago de Compostela, Spain

## Abstract

Oncogene-induced senescence (OIS) is a complex process, in which activation of oncogenic signals during early tumorigenesis results in a high degree of DNA replication stress. The ensuing response to the DNA damage produces a permanent G1 arrest that prevents unlimited cell proliferation and lessens the development of tumours. However, despite the role of OIS in the proliferative arrest resulting from an activating oncogenic-lesion has obtained wide support, there is also evidence indicating that cells may overcome oncogene-induced senescence under some circumstances. In this study, we have investigated the possibility that some of the assumptions on the role of DNA damage response (DDR) in triggering OIS may depend on the fact that most of the available data were obtained in mouse embryo fibroblast. By comparing the degree of OIS observed in mouse embryo fibroblasts (MEF) and mouse embryo astrocytes (MEA) obtained from the same individuals we have demonstrated that, despite truthful activation of DDR in both cell types, significant levels of OIS were only detected in MEF. Therefore, this uncoupling between OIS and DDR observed in astrocytes supports the intriguingly possibility that OIS is not a widespread response mechanism to DDR.

## Introduction

Cellular senescence is a permanent G1 arrest of cell proliferation that limits the lifespan of mammalian cells and prevents unlimited cell proliferation. Cellular senescence may be triggered by numerous cell intrinsic and extrinsic mechanisms such as the progressive telomere attrition produced by cell replication (replicative senescence), or the response to multiple adverse stimuli such as oxidative stress or DNA damage induced by oncogenic stimuli (oncogene-induced senescence, OIS). While replicative senescence is largely linked with the aging process or the pathogenesis of some degenerative diseases, OIS should be considered as a potent defensive mechanism against cancer, since once senescence is activated cells are not capable of developing into tumours^1,2^.

Although OIS is a complex process in which multiple signalling proteins network to trigger the response, accumulating evidence suggests that all of them operate via RB and p53^1^. Activation of oncogenic signals during early tumorigenesis leads to an unregulated stimulation of growth promoting genes which results in a high degree of DNA replication stress that produces numerous double-stranded DNA breaks. The ensuing DNA damage response (DDR) drives the activation of several tumour suppressor networks, including the p16^INK4A^/RB and p19^ARF^/p53/p21 pathways^3,4^ which, in turn, trigger OIS, thereby arresting cells within a few cell-division cycles after oncogene expression^5^.

However, despite a plethora of reports on the role of OIS on the proliferative arrest resulting from an activating oncogenic-lesion^5,6^, there is also evidence indicating that cells may overcome OIS under some circumstances^7–11^. Although the reasons for this different response are still under debate, several factors such as effect of signal intensity or the cell type investigated have been proposed. Since most of the studies supporting the dogma that OIS is a necessary consequence of DDR have been performed in mouse embryo fibroblasts (MEF), in this report we have investigated the differential senescence response in MEF as compared with mouse embryo astrocytes (MEA) obtained from the same individuals. Our results indicate that despite a similar degree of DNA damage, OIS response is clearly different between MEF and MEA, thus suggesting that some of the assumptions on the mechanisms of OIS and its biological implication should be revised.

## Results

**Figure 1:**
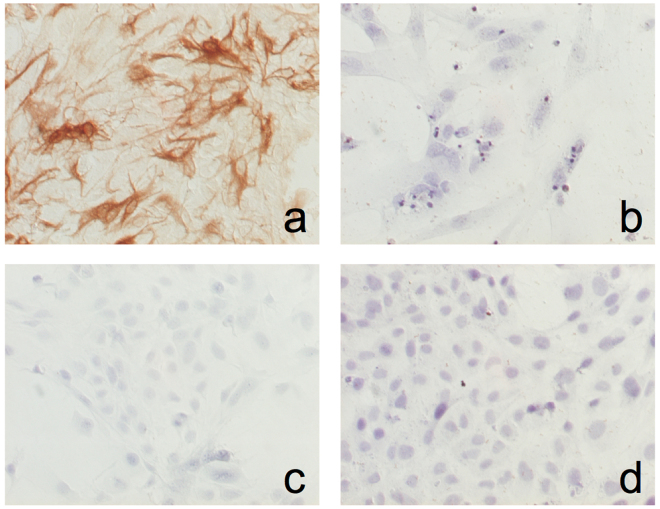
Characterization of MEA cultures. (**a**) MEA were incubated with a polyclonal anti-GFAP antibody, and GFAP immunoreactivity was detected using the avidin-biotin complex for amplifying the target antigen signal, as indicated in materials and methods. (**b**) Negative control (without anti-GFAP antibody). (**c**) MEA were incubated with a polyclonal anti-vimentin antibody. (**d**) MEF were incubated with the monoclonal anti-GFAP antibody. In both cases, antigen signal was amplified as indicated above.

Wild type (control) primary MEA showed a flat, polygonal shape, with few projections and non-retractile cytoplasm. Immunostaining revealed that more than 95% of the cells were positive for GFAP, while no vimentin staining was detected, thus ruling out the presence of mesenchymal cells in the culture. In contrast, no GFAP staining was detected in MEF (Fig. 1).

**Figure 2:**
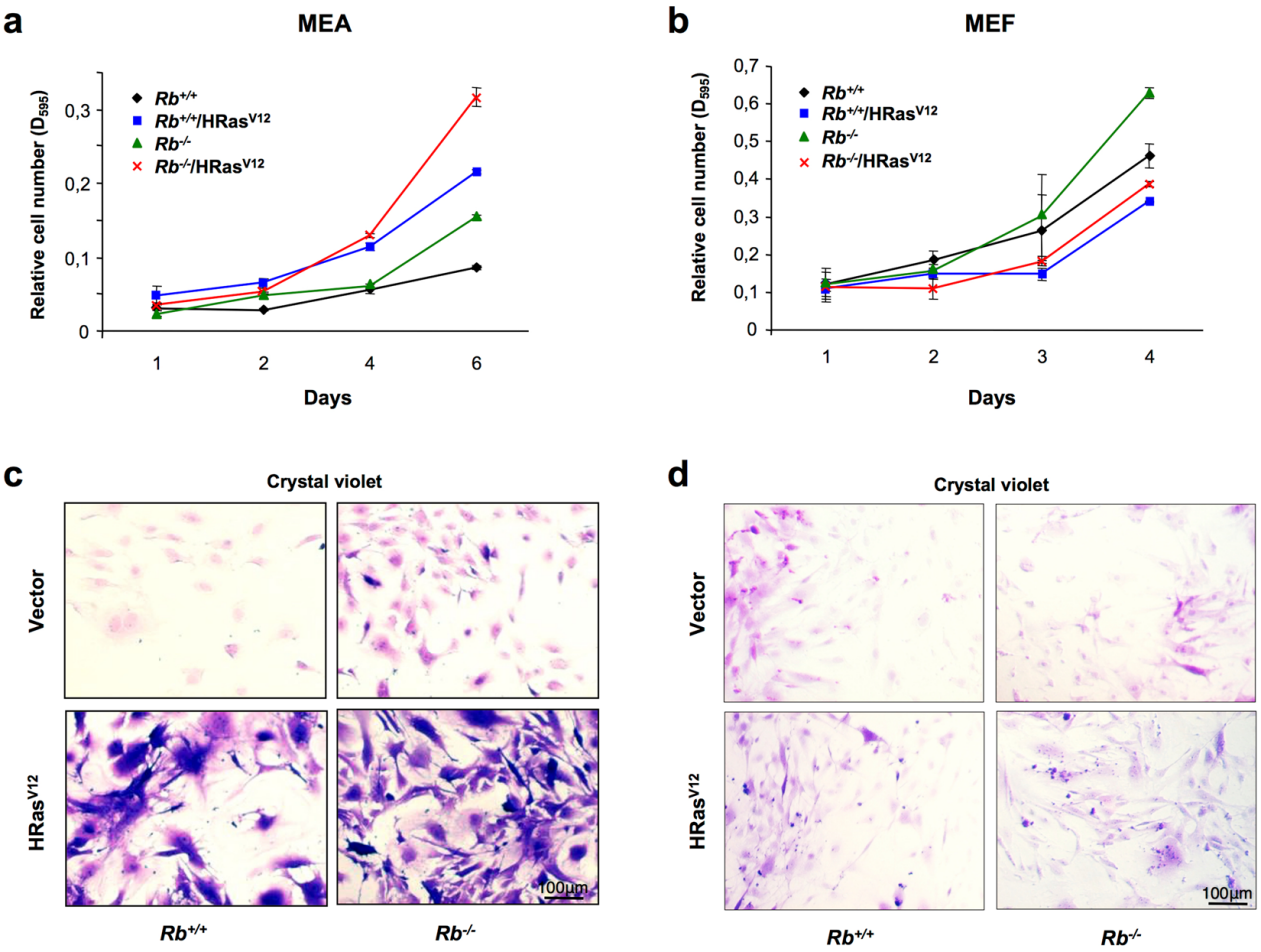
Overexpression of HRas^V12^ induces cell cycle arrest in MEF but not in MEA. (**a**,**b**) Growth curve analysis of *Rb* ^+/+^ or *Rb* ^−/−^ MEA (**a**) or MEF (**b**) infected with pBABE or pBABE-HRasV12 retroviral vectors. After infection, cells were plated in triplicate and the cells were fixed on the indicated days for subsequent staining with crystal violet. Each time point represents the mean ± S.D. of total cumulative cell number from at least three independent experiments. (**c**,**d**) Representative images of results presented in (**a**,**b**), respectively. All photographs are at the same magnification.

Control MEA displayed a clearly lower growth rate when compared with MEF: a two-fold increase in the relative cell number was observed in MEA after 6 days in culture, while MEF experimented a 4-fold increase in only 4 days (Fig. 2a and b). Similarly, while lack of RB increased the growth rate in both MEA and MEF, this increase was more evident in the latter (Fig. 2a and b). Interestingly, despite this increased proliferative rate, no morphological differences were found between *Rb* ^+/+^ and *Rb* ^−/−^ cells in either MEA or MEF (Fig. 2c and d).

On the other side, ectopic expression of oncogenic Ras resulted in opposite effects in MEA and MEF growth. In keeping with previous reports^12,13^, expression of oncogenic Ras reduced the growth rate in both *Rb* ^+/+^ and *Rb* ^−/−^ MEF, an effect that may be consequence of premature OIS. At odds with this, a dramatic increase in the growth rate was observed when HRas^V12^ was expressed in MEA and, intriguingly, the highest growth rate was observed when oncogenic Ras was expressed in MEA lacking RB. Furthermore, along with these differences in the growth rate, prominent differences in the morphological characteristics were also detected between MEA and MEF. While no appreciable morphological changes were observed in MEF overexpressing HRas^V12^ (Fig. 2d), HRas^V12^-expressing MEA showed dramatic morphological changes and, although some flat polygonal cells were still present, most of MEA displayed very large cytoplasm with long thin projections (Fig. 2c). Although these phenotypic changes were observed in both *Rb* ^+/+^ and *Rb* ^−/−^ MEA, there were much more obvious in the latter. The intense morphological changes suggest the existence of a transformed phenotype, in fact *foci* growth was induced by HRas^V12^ expression in either *Rb* ^+/+^ and *Rb* ^−/−^ postnatal astrocytes. Indeed, postnatal astrocytes expressing HRas^V12^ are also able to form detectable tumours within 8–15 weeks whereas tumours formed by *Rb* ^−/−^/Ras^V12^ appeared in-between the first and second week^14^.

**Figure 3:**
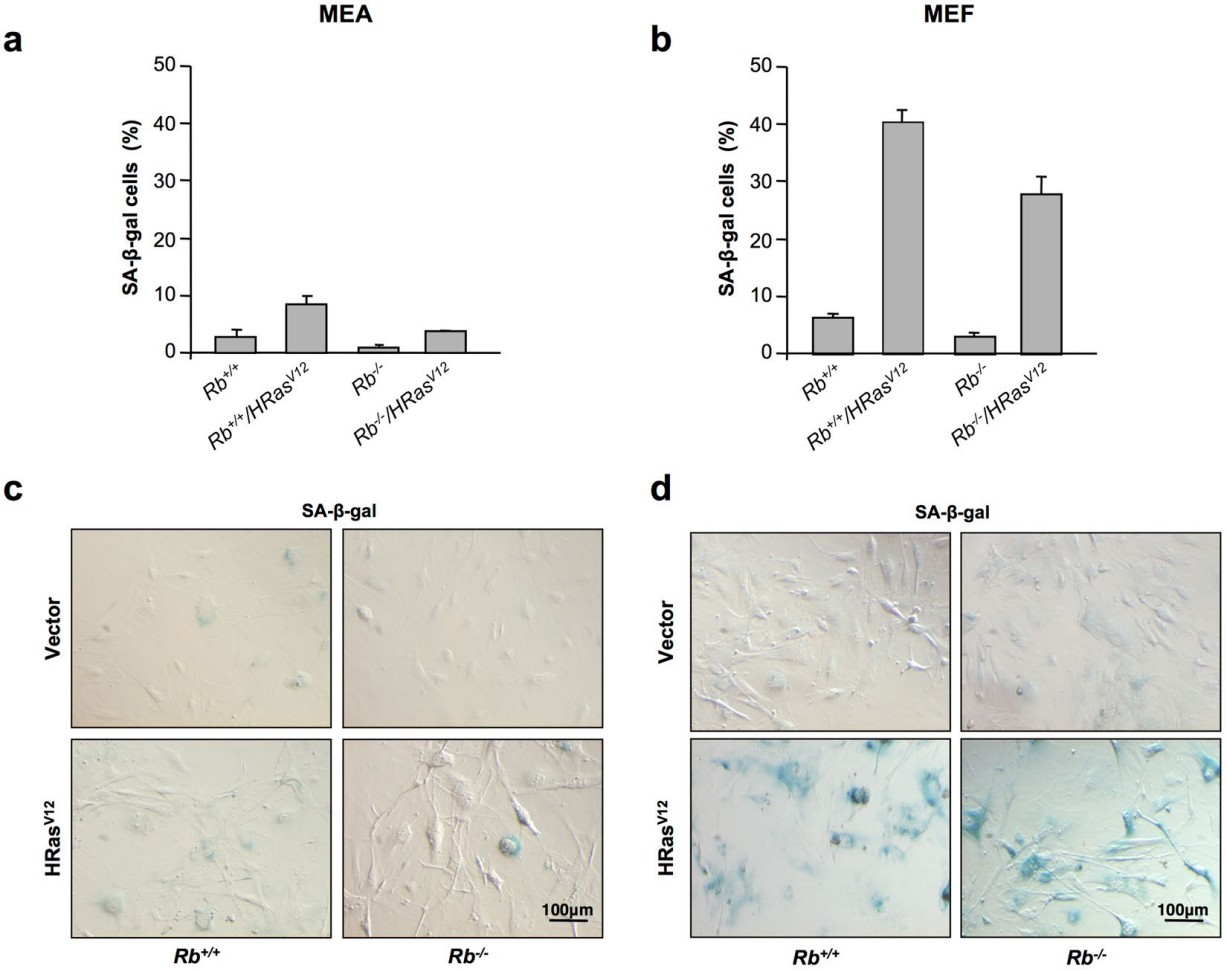
Overexpression of HRas^V12^ induces senescence in MEF but not in MEA. (**a**,**b**) Senescence assays in *Rb* ^+/+^ or *Rb* ^−/−^ MEA (**a**) or MEF (**b**) infected with pBABE or pBABE-HRas^V12^ retroviral vectors. The y-axis represents the percentage of SA-β-galactosidase-positive cells (mean ± S.D.) from at least three independent experiments. (**c**,**d**) Representative images of results presented in (**a**,**b**), respectively. All photographs are at the same magnification.

Altogether, these different effects on cell growth and morphology suggest the existence of a different response to oncogenic stress between MEF and MEA. Therefore, to further investigate this possibility, we investigated the effect of oncogenic stress on cellular senescence in both cell types. As previously mentioned, OIS is part of the DDR and, in keeping with this, β-gal staining revealed a clear-cut increase in the number of senescent cells in MEF expressing oncogenic Ras. In contrast, the number of senescent cells was barely increased when oncogenic Ras was expressed in MEA (Fig. 3). Lack of *Rb* did not clearly modify the outcome of cells overexpressing HRas^V12^ in either MEF or MEA, although a slight decrease in the number of senescent cells was observed in both cases.

**Figure 4:**
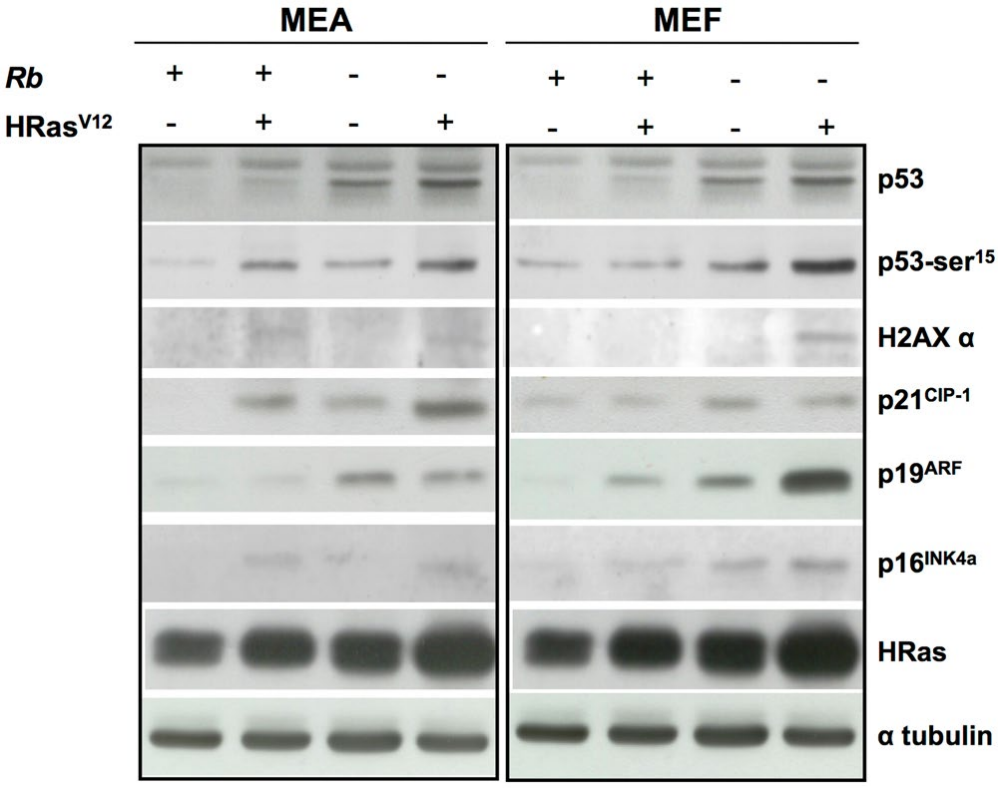
Uncoupling of the DDR from OIS in MEA but not in MEF. Immunoblot analysis of DNA damage checkpoint response performed on lysates prepared from *Rb* ^+/+^ or *Rb* ^−/−^ MEA or MEF infected with pBABE or pBABE-HRas^V12^ retroviral vectors (5 and 4 days, respectively, after cell were seeded).

Finally, and to elucidate the mechanisms underlying this different response to OIS between MEA and MEF, we explored its effect of some of the well-characterized indicators of DDR in both cell types. As Fig. 4 depicts, Ras activation in MEF induced the accumulation of both p53 and p-p53^Ser15^, together with α-H2AX and p21^CIP1^. In all, these findings suggest the existence in these cells, as expected, of a DNA damage induced by Ras-driven DNA hypereplication. This effect was more evident in cells from *Rb* ^−/−^ mice, which probably reflects the fact that increased E2F activates p19^ARF^ which, in turn, inhibits MDM2, thus favouring p53 accumulation. Interestingly, despite there is strong evidence indicating that activation of p53 is related to the induction of cell senescence, we were unable to find any correlation between the degree of activation of this pathway and the extent of cell senescence. Thus, while senescence levels were slightly greater in cells from *Rb* ^+/+^ mice than in their *Rb* ^−/−^ counterparts, the higher activation of p53 pathway was observed in *Rb* null cells.

However, the lack of correlation between biochemical and biological outcomes was more clearly evidenced in MEA. As previously stated, cellular senescence was barely present in these cells, even after activation of oncogenic Ras. Intriguingly, this lack of OIS was observed in cells that accumulate p53, p-p53^Ser15^, α-H2AX and p21^CIP1^, thus indicating the existence of a characteristic DDR response, similar to that observed in MEF. As occurred in MEF, the greatest activation of OIS checkpoints was observed in MEA obtained from *Rb* ^−/−^ HRas^V12^ mice, but also in this case, the correct activation of DDR was unable to induce cellular senescence.

## Discussion

OIS is as a permanent state of proliferative arrest resulting from an activating oncogenic-lesion that has been suggested to function as a cancer cell intrinsic mechanism to restrain tumour growth. However, albeit the role of OIS on the induction of proliferative arrest in response to an activating oncogenic event is broadly recognised^5,6^, there is also evidence indicating that cells may overcome OIS in some situations. In fact, in this report, we demonstrate that MEA are resistant to Ras-induced senescence despite adequate DDR activation, thus suggesting that OIS may be absent in these cells.

OIS was first evidenced as a senescence-like phenotype associated with overexpression of activated Ras proteins^12^, but has been associated to ectopic expression of other oncogenes since that^15–17^. In mice, Ras-driven senescence is mediated by activation of p19^ARF^ which detects the oncogenic signal and triggers the phosphorylation of p53 tumour suppressor. Activation of p53 induces the expression of the cell cycle inhibitor p21, that causes growth arrest^12,18^. Consistent with this, overexpression of activated K-Ras does not trigger senescence in fibroblasts derived from p19^ARF^ null mice^19^. In addition, DDR also induces expression of the CDK inhibitor p16^INK4A^, a tumour suppressor that blocks the inhibitory phosphorylation of RB^20–22^. Subsequent activation of RB silences the transcription of several E2F-dependent genes required for G1/S progression, thereby contributing to the arrest of cell proliferation^23–25^. However, p16^INK4A^ null primary mouse fibroblasts remain susceptible to OIS^26,27^, thus suggesting that, at odds with p19^ARF^_,_ this protein plays a minor role in promoting OIS, at least in those cells. In keeping with this, lack of RB barely modifies the outcome of OIS after Ras activation in either MEF or MEA.

Although this counter-intuitive concept that activation of oncogenes leads to growth arrest has become one of the mainstays of OIS, at least in mice, there is also evidence indicating that Ras-driven senescence can be bypassed under several conditions. Thus, in contrast with MEF response to the overexpression of oncogenic H-Ras, MEF expressing a single activated K-Ras allele are immortal^9^, and, similarly, inactivation of NF1 (which encodes a RasGAP protein) induces an activation of endogenous Ras that results in MEF immortalization^10^. Finally, H-Ras triggered senescence can be observed in mammary epithelial cells when high levels of activated Ras are present, while low levels of Ras may even trigger tumour formation if senescence checkpoints are inactivated^12^.

Although these findings support that signal intensity may play a key role in mediating a senescence response, there is also evidence indicating that other factors such as the cell type or even the species may be the actual matter. Thus, at odds with mice, p14^ARF^ (the human homolog of p19^ARF^) does not show a so straightforward relationship with OIS, and although Ras-induced senescence has been reported, there is also evidence indicating that human fibroblasts may be resistant to Ras-driven senescence^28^. Similarly, HRas^V12^ has been also demonstrated to induce OIS independently of p53 in primary human oesophageal keratinocytes, while depletion of p53 allows the proliferation of HRas^V12^-expressing normal human fibroblasts^5,29^.

On this regard, we have found in the present study that, despite DDR is similar in MEA and MEF, senescence response is only observed in the latter. It must be taken in consideration that both MEA and MEF were obtained from the same animals and cultured under the same conditions, thus ruling out any influence of strain or genetic background, as well as other methodological pitfalls. To the present, MEF have been widely used to study many cellular processes such as cell growth, survival, differentiation or senescence, and have served as a helpful tool to recognize regulatory molecules involved in neoplastic transformation^12,19,30–32^. However, on the view of our results, we propose that some of the assumptions that have been originated from MEF, cannot be straightforwardly extended to other cell types. Therefore, it is tempting to speculate that OIS mechanisms in MEF may reflect the low incidence of tumours in mesenchymal cells. On the contrary, ability of MEA to escape cellular senescence upon activation of Ras and elimination of RB could underlie the higher incidence of epithelial cancer. Further research will be needed in order to support the actual extent of this proposal.

In summary, the results presented in this study also support the view that Ras activation is not unavoidably linked to cell senescence even in mice, thus supporting the intriguingly possibility that OIS is not a widespread mechanism linking DDR and cellular senescence.

## Methods

Primary monolayer cultures of either MEF or MEA were established from 13.5 dpc (day post coitum) *Rb* ^−/−^ or *Rb* ^+/+^ mice. The care and use of all experimental animals was in accordance with institutional guidelines and approved by the Ethics Committee of the University of Santiago de Compostela and Xunta de Galicia (approval ID 15005AE/07/FUN01/FIS02/JACP1). Both cell types were plated in Falcon polystyrene culture dishes (BD Biosciences, Madrid, Spain) and grown in Dulbecco’s modified Eagle’s medium (DMEM, Sigma-Aldrich, Madrid, Spain) supplemented with 10% foetal bovine serum (Thermo Fisher Scientific, Madrid, Spain), 2 mM glutamine, 2.5 U/mL penicillin, 2.5 μg/mL and streptomycin (all from Thermo Fisher Scientific). The cultures were maintained at 37 °C in a humidified atmosphere of 5% CO_2_.

Pregnant mice were sacrificed at 13.5 dpc. Each embryo was transferred to individual petri dish with DMEM. Once red organs were dissected, the head and carcass of each embryo were individually transferred to new dry petri dishes for isolation and culture of MEA and MEF, respectively, and head was used to perform culture.

MEF cultures were performed as previously described^33^. Briefly, each chopped embryo carcass was resuspended in 5 mL of cold PBS and transferred into a 25 mL falcon tube and settled for 5 min. Supernatant was discarded and the process was repeated twice. PBS was replaced with 2 mL of cold trypsin/EDTA 0.05% (Thermo Fisher Scientific) and the cells were incubated at 4 °C for 6 to 18 hours, followed by incubation a 37 °C for 20 min. After 5 min of incubation, cells were dissociated by pipetting up and down thoroughly, and then trypsin was inactivated by adding 2 volumes of complete medium.

Dissected heads were used for MEA culture as previously described, with appropriate modifications^34^. Each head was placed in a fresh petri dish with 1 mL of DMEM, and a transverse incision was performed in the forehead line. The brain was gently pushed out of the skull through the incision and, after removal of olfactory bulbs and cerebellum, was dissociated by mechanical shearing with a p1000 pipette in 1–2 mL of fresh medium. The cell suspension was filtered through a 40 µm cell strainer (Millipore) with the help of the syringe plunger to obtain a total disaggregation of the cells. Then, the cell strainer was washed with 10 mL of DMEM and the cells were centrifuged for 5 min at 300 × g. Supernatant was carefully removed and the cells were resuspended in 10 mL of complete medium. To remove overlaying microglia and oligodendrocyte precursor cells^34,35^, cells underwent a harsh washing process entailing at least five rounds of intensive rinse with PBS followed by vigorous shaking of the culture dish. Cells were then cultured for 24 h and the washing process was repeated. Finally, cells were trypsinized and plated in the appropriate concentration.

### Retroviral Transduction

For introduction of an activated Ras allele into MEA or MEF, cells were transduced with pBABE-puro-empty or pBABE-puro-HRas^V12^ retroviral vectors (a gift from P.P. Pandolfi). Retrovirus Production and Infection were carried out using Phoenix-Eco packaging cells (a gift from G.P. Nolan) as previously described^36^. For each cell type (MEF or MEA), four experimental groups were established: control (*Rb* ^+/+^), *Rb* ^−/−^, *Rb* ^+/+^-HRas^V12^ and *Rb* ^*−*/*–*^HRas^V12^. Infection of MEF and MEA was carried out simultaneously using the same supernatant from transfected Phoenix cells. After retroviral infection MEF and MEA were selected with 2 μg/mL of puromycin for 3 to 4 days before carrying out the experiments.

### Proliferation assay

To measure proliferation, cells were seeded at low confluence in 24-well plates (1*10^5^ MEF or MEA per well) in triplicate and fixed in cold methanol for 15 min on indicated days for subsequent staining with crystal violet (0.05%) for 30 min. After washing, the cellular content of crystal violet was extracted with 10% acetic acid, and the relative cell number was calculated based on the absorbance levels at 595 nm.

### Immunocytochemistry

MEA were characterized in culture by immunocytochemistry using a monoclonal antibody against glial fibrillary acidic protein (GFAP; Agilent Technologies, Madrid, Spain; dilution 1:400) and a polyclonal anti-vimentin antibody (Abcam, Cambridge, MA; dilution 1:500). GFAP or vimentin immunoreactivities were detected using the EnVision Detection System (Agilent Technologies) according with the manufacturer’s instructions. MEF were used as negative controls.

### SA-β-galactosidase assay

SA-β-galactosidase activity was assessed with the senescence β-galactosidase staining kit (Werfen, Barcelona, Spain) according with the manufacturer’s instructions.

### Western blot

Cells were collected by centrifugation, washed twice in ice-cold PBS and lysed in ice-cold RIPA buffer (1x PBS, 1% Nonidet P-40, 0.5% sodium deoxycholate, 0.1% SDS, 10 mg/mL PMSF, 40 mg of aprotinin/mL, 100 mM orthovanadate) (all reagents from Sigma-Aldrich). After centrifugation (15000 g, 15 min, 4 °C) to separate cellular debris, the lysates were resolved in a 12% SDS-PAGE, and electrotransferred onto a nitrocellulose paper (Protran; Schleicher and Schuell, Dassel, Germany). Determination of protein levels was carried out by immunoblot analysis using antibodies against p53 (CM5; Leica Biosystems, Barcelona, Spain), p-p53^S^ ^er^ ^1^ ^5^ (9284; Werfen), p-H2AX Ser139 (07–164, EMD Millipore, Madrid, Spain), p19ARF (ab80; Abcam), p16 (M-156; Santa Cruz Biotechnology, Heidelberg, Germany), pan-Ras-V12 (Ab-1; Sigma-Aldrich) and β-tubulin (T5168; Sigma-Aldrich).

### Data Availability

No datasets were generated or analysed during the current study.
